# Consumer Acceptance toward Functional Foods: A Scoping Review

**DOI:** 10.3390/ijerph19031217

**Published:** 2022-01-22

**Authors:** Mathew T. Baker, Peng Lu, Jean A. Parrella, Holli R. Leggette

**Affiliations:** Department of Agricultural Leadership, Education and Communications, Texas A&M University, College Station, TX 77843, USA; peng.lu@ag.tamu.edu (P.L.); Jparrella@tamu.edu (J.A.P.); Holli.Leggette@ag.tamu.edu (H.R.L.)

**Keywords:** functional foods, consumer acceptance, scoping review

## Abstract

Chronic diseases (e.g., heart disease, cancer, diabetes) are of major public concern. Such chronic diseases are often caused by a dietary pattern characterized as relatively high in fat, refined sugar, salt, and cholesterol. Societal interest in consuming healthy foods and the demand for healthy food products have increased significantly. As a result, functional foods have gained significant research attention in the food health and technology innovations field. To date, many studies have investigated the factors that may predict consumer acceptance of functional foods, and a wide range of influential factors have been reported. However, studies conducted in different contexts pose challenges to gaining a clear understanding of the factors influencing consumer acceptance. Therefore, the purpose of our scoping review was to synthesize the possible determinants of consumer acceptance toward functional foods and provide a resource that describes global trends regarding consumers’ functional foods behavior. We identified 75 articles published with varying populations around the globe that empirically investigated consumers’ acceptance of functional foods. We identified and categorized a wide range of determinants related to consumer acceptance of different types of functional foods. The five categories of determinants were product characteristics, socio-demographic characteristics, psychological characteristics, behavioral characteristics, and physical characteristics. Each of the determinants were more fully described by sub-determinants in our scoping review. These determinants should be considered and used by leaders and scientists in product development to aid decision making and, ultimately, the successful launch of novel functional foods.

## 1. Introduction

Chronic diseases (e.g., heart disease, cancer, and diabetes) are of major public concern. Such chronic diseases are often caused by a dietary pattern characterized as relatively high in fat, refined sugar, salt, and cholesterol [1]. Older persons are at an increasingly higher risk of developing chronic diseases, which is becoming a significant problem as the world population continues to age [2]. In addition to an aging population and the increased development of chronic diseases, the steady increase in life expectancy and quality coupled with severe side effects caused by drugs and pharmaceuticals have driven the need for developing safety-affirmed foods enriched with adequate nutrients [3,4]. Consuming foods enriched with functional ingredients (e.g., vitamins, probiotic, minerals, fiber, and antioxidants) could reduce the risk of chronic diseases and improve physical and mental well-being [5,6,7].

Functional foods have gained significant research attention throughout the decades, especially in the areas of improved food health and technology [3]. The concept of functional food has been defined several times, yet there is no universally accepted definition of this term [8,9,10]. Between 1995 and 1998, more than 100 experts in nutrition and related sciences reached a consensus on the definition of functional foods as part of the European Commission’s Concerted Action on Functional Food Science, coordinated by the International Life Sciences Institute. The European Consensus Document stated that “food can be regarded as functional if it is satisfactorily demonstrated to affect beneficially one or more target functions in the body, beyond adequate nutritional effects, in a way that is relevant to either improved stage of health and well-being and/or reduction of risk of disease” [11] (p. 6), which has been the most widely cited definition of functional food in previous studies.

Alternative definitions also exist across food and nutrition institutes. For example, in 1994, the Institute of Medicine of the U.S. National Academy of Sciences’ Food and Nutrition Board defined functional foods as “any modified food or food ingredient that may provide a health benefit beyond the traditional nutrients it contains” [12] (p. 109). Similarly, in 2005, the Institute of Food Technologists defined functional foods as foods that provide a health benefit beyond their basic nutrition [13]. More recently, in 2014, the Functional Food Center defined functional foods as “natural or processed foods that contain biologically active compounds; which, in defined, effective, and non-toxic amounts, provide a clinically proven and documented health benefit utilizing specific biomarkers for the prevention, management, or treatment of chronic disease or its symptoms” [14] (p. 215). However, despite these varying definitions, experts generally agree that functional foods contain ingredients that provide health benefits beyond the food’s basic nutritional components.

With the popularity of functional foods, people are becoming increasingly aware of food quality and the health benefits associated with different foods [4]. As a result, people’s interest in consuming healthy foods and the demand for healthy food products have increased significantly. Therefore, it is necessary to develop novel functional foods to meet these demands [15]. However, not only is the development of functional foods a complex and expensive process that involves uncertainty and risk issues, but consumers’ food adoption is also a complex and slow process that is dependent on many factors [16]. Because consumers’ uncertainty and skepticism toward novel functional foods could influence their acceptance of such products [10,17,18], understanding their responses to functional foods is vital [10].

To date, many studies have investigated the factors that may predict consumer acceptance of functional foods, and a wide range of influential factors have been reported. However, studies conducted in different contexts pose challenges to gaining a clear and comprehensive understanding of the factors influencing consumer acceptance. The variety of factors and the complex relationships between them make it difficult to describe general trends, which would benefit scientists and functional food manufactures when developing and launching functional foods. The wide range of influential factors also poses challenges for communicating and marketing professionals in the functional foods industry when developing accurate and precise communications strategies and other promotional materials designed to improve consumers acceptance of functional foods.

Reviews published in the context of healthy or modified foods have focused on consumer evaluation of food with nutritional benefits [19], the credibility of functional product effects [20], nutrition-modified and functional dairy products [21], and organic food consumption [22]. Together, these reviews have provided some valuable insights into the factors influencing consumer acceptance of healthy foods or specific functional food aspects. However, to the best of our knowledge, there has been no scoping review that comprehensively synthesizes the identified factors that may predict consumer acceptance of functional foods. Therefore, the purpose of our scoping review was to synthesize the possible determinants of consumer acceptance toward functional foods and provide a resource that describes global trends regarding consumers’ behaviors toward functional foods.

## 2. Methods

A scoping review can be conducted to systematically explore the literature, synthesize existing evidence, and address knowledge gaps [23]. Therefore, a scoping review was the most appropriate methodology to achieve the study’s purpose because it allowed us to gather relevant literature across databases, identify and synthesize key factors influencing consumer acceptance of functional foods, and develop a novel comprehensive understanding of this phenomenon. Our scoping review was conducted by following the guides developed by Arksey and O’Malley [24]. The procedures were as follows: to identify research objectives, to identify databases, to develop search strategies, to determine inclusion and exclusion criteria to screen relevant studies, to develop a coding approach to categorize determinants, and to summarize and report the results.

### 2.1. Research Objectives

To achieve the study’s purpose of synthesizing possible determinants of consumer acceptance toward functional foods and providing a resource that describes global trends, the three objectives were as follows: (1) to outline the included studies’ characteristics, (2) to identify and categorize the determinants investigated in reviewed studies, and (3) to provide implications for future social and behavioral scientists who work in the domain of consumer acceptance of functional foods. We believe this review will benefit leaders and researchers in product development by providing comprehensive evidence that aims to improve consumer acceptance of functional foods.

### 2.2. Databases and Search Strategy

We conducted literature searches in October 2020 using four databases—Web of Science Core Collection, Medline (OVID), CAB abstracts, and Google Scholar. We selected these databases after consulting with a subject librarian. Using these four databases ensured the adequate inclusion of relevant references in our scoping review. We began the search process using Google Scholar, which uses a full-text indexes approach. Therefore, we reviewed the first 200 search results presented by Google Scholar, which the database deemed most relevant based on our search terms. After reviewing these search results from Google Scholar, we determined the search terms and Boolean operators for the other three databases (Web of Science Core Collection, Medline [OVID], and CAB Abstracts). The first set of search terms included “functional food*” OR “functional product*” OR “enriched food*” OR “enriched product*” OR “fortified product*”. The second set of search terms included “consumer accept*” OR “consumer purchase behavior*” OR “consumer attitude*” OR “consumer perception*” OR “consumer willingness to pay” OR “consumer willingness to buy”. Then, we conducted a manual search for several additional studies that we obtained from the reference lists of studies already included. We validated our search process by examining reviews published on similar topics and comparing their included studies and reference lists to ours.

### 2.3. Study Management and Screening

Covidence systematic review management software was used for the management of the published research and subsequent screening. The established inclusion and exclusion criteria that informed the process of screening are presented in Table 1.

**Figure 1:**
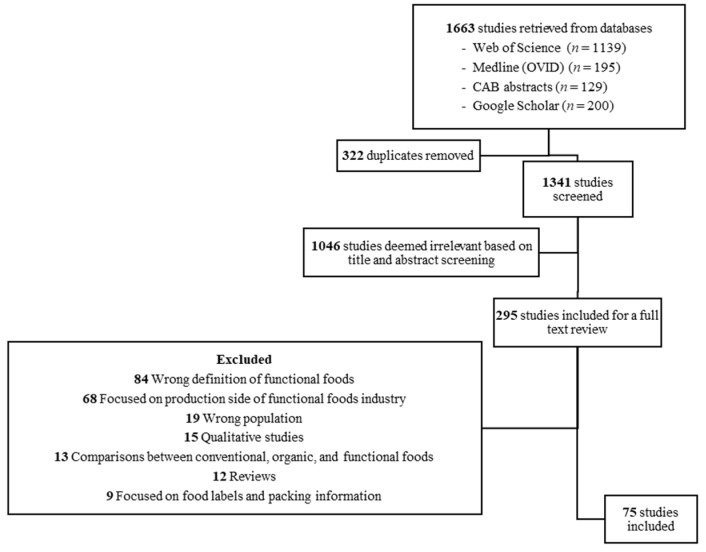
Literature review search and screening process.

First, the identified studies were assessed through title and abstract screening. Then, a full-text review was conducted to identify studies that satisfied all inclusion criteria. Two of the authors independently completed the title and abstract screening of the initial 1341 studies (after duplicates were removed) and achieved an agreement rate of 89.41% (*n* = 1199). We solved the discrepancies through discussions and consultations with the third and fourth authors until consensus was reached [23]. Ultimately, 75 studies were included in our scoping review (see Figure 1).

### 2.4. Data Extraction and Determinants’ Synthesis

Data extraction was performed using a data extraction template developed by the authors based on our research objectives. The data extraction template included the following categories: authors and year of publication, research method, functional foods studied, continent in which the research was conducted, sample size, key findings, determinants, and outcome measures (see Appendix A). Two authors independently extracted this information from the 75 included studies. We used open coding procedures to extract the included studies’ characteristics and classify the determinants of consumer acceptance of functional foods into five broad categories. The categories of the determinants were based on our modification of a previously published review [21].

## 3. Results

**Figure 2:**
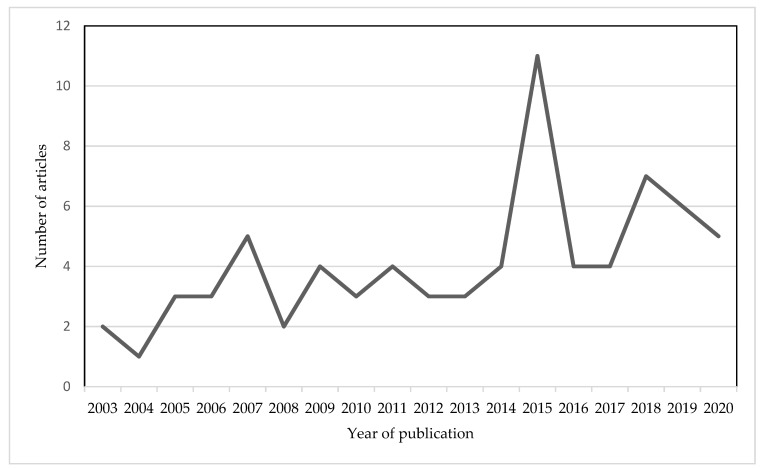
Year of publication of the 75 articles included in the scoping review.

Most of the 75 included studies were conducted in Europe (*n* = 47) and Asia (*n* = 17), with 11 studies conducted in North America (*n* = 6), South America (*n* = 1), and Australia/Oceania (*n* = 4) published between 2000 and 2020 (see Figure 2). Among the studies included, surveys were the most common research method used to assess consumer acceptance of functional food. The data collection strategies and research methods varied, and included face-to-face questionnaires [25,26], computer-aided questionnaires [27,28,29], questionnaire-based economic evaluation techniques such as experimental auctions [30,31,32], conjoint analysis [33,34,35], and choice experiments [36,37]. The types of functional foods investigated in the included studies were functional meats, functional beverages, functional dairy products, functional fruits, and functional snacks (e.g., cookies, yogurt, and cereals).

**Figure 3:**
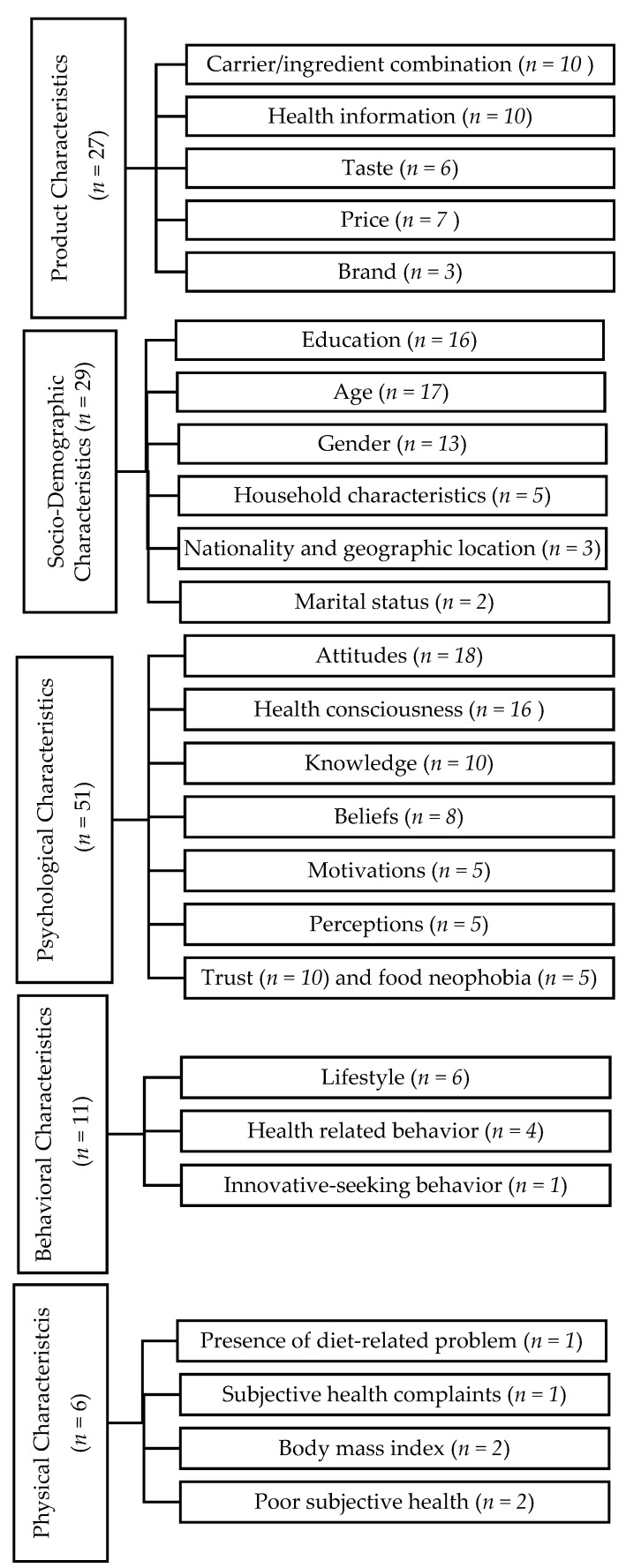
Categories and determinants of consumer acceptance toward functional foods and the number of articles that were investigated.

Based on our consensus, the determinants were classified into five categories. The five categories—product characteristics, socio-demographic factors, psychological characteristics, behavioral characteristics, and physical characteristics—are described below. Each of the categories are more fully described by individual determinants (see Figure 3). Consumer acceptance is defined as outcome measurements in our scoping review, which includes general acceptance [38], willingness to pay [30,37], willingness to buy [39,40], willingness to try [33], perceptions of functional foods [34,41], consumption of functional foods [42,43], purchase intention [44,45,46,47], and choice of functional foods [48,49,50].

### 3.1. Product Characteristics

The reviewed studies indicated that product characteristics (i.e., the combination of carriers and ingredients, price, taste, brand, and health information) can influence consumer acceptance of functional foods.

#### 3.1.1. The Combination of Carriers and Ingredients

Functional foods are those fortified with vitamins, minerals, and various micronutrients [51], and adding new functional ingredients to a functional food carrier is how new functional foods are developed [52]. For example, yogurt (carrier) can be enriched with antioxidants and fiber (functional ingredients; [48]). The combination of carriers and ingredients used to create functional foods has been identified as a critical factor influencing consumers’ perceptions and acceptance of the products [28,53,54,55,56]. Previous studies found consumers were more likely to accept functional foods with perceived healthier carriers and natural enrichments. For example, participants in Van Kleef et al.’s [57] study showed greater preferences for healthier carriers (e.g., margarine and yogurt) when compared with indulgence-type foods (e.g., chewing gum, ice cream, and chocolate). Similarly, Verbeke et al. [58] found fiber-enriched cereals were more accepted than calcium-enriched juice because of the less healthy combination of juice with calcium. These findings were consistent with Bech-Larsen and Grunert’s [59] findings that consumers considered inherently wholesome foods (e.g., yogurt) as being healthier carriers than unwholesome foods (e.g., spreads).

In addition to the influence of functional carriers, the manner in which the product ingredients were manufactured or inserted affected consumer acceptance. Specifically, consumers were inclined to accept functional foods in which the ingredient enrichment process occurred naturally [46,53]. Jahn et al.’s study [46], which measured consumers’ perceived appropriateness of functional food carriers, suggested that less processed products (e.g., milk) were considered more natural and appropriate for vitamin D fortification compared to processed products (e.g., sausage, fish, and liver pate). Furthermore, in an unrelated study, Krutulyte et al. [55] found that consumers tend to be more accepting of functional foods that contain an ingredient/carrier combination with which they are already familiar.

#### 3.1.2. Price

Previous studies have found that the price of functional foods may have some influence on consumer acceptance [48,60,61]. In general, consumers tend to pay a reasonable price to get the health benefits of consuming functional foods [45,61,62]. Accordingly, price could affect consumer acceptance in two contradicting ways: (1) a higher price may decrease consumers’ purchase intention or (2) a higher price may increase consumers’ purchase intention because it may increase the products’ perceived quality [45,48,63]. Ares et al. [48] found that price had a significant negative effect on consumers’ consumption of functional yogurts. Similarly, Narayana et al. [50] found many Sri Lankan consumers were more concerned about the price of functional foods rather than the health benefits associated with consuming them. However, Ares et al. [48] argued that if consumers were more interested in combatting health issues, they could be less sensitive to a higher price. Huang et al. [45] similarly explained that a negative effect of price on consumer acceptance could be counteracted by health consciousness. For example, consumers who showed a higher concern for their personal health were willing to pay more for the health benefits associated with consuming functional foods [31]. However, it should be noted that, in certain cases, consumers were only willing to pay a limited premium price. For example, Mirosa and Mangan-Walker [61] found that Chinese consumers were not willing to pay more than 40% extra for functional foods, and Menrad [64] found that European consumers would only pay 30–50% extra for functional foods.

#### 3.1.3. Taste

The effect of taste on consumer acceptance has received considerable attention in previous studies. Taste or expected taste strongly influences consumers’ functional food choices [30,47]. For example, a study conducted in 2020 by Narayana et al. [50] found that taste was one of the most important motives for consuming functional foods among Sri Lankan consumers. In many cases, the influence of taste might surpass the influence of health benefits [65] as several studies have confirmed consumers’ unwillingness to compromise on taste for health benefits [28,65,66]. As Verbeke [67] argued, it is highly risky to assume that consumers would accept functional foods that are not tasty.

#### 3.1.4. Brand

Previous studies have also found that brand can strongly influence consumers’ functional food choices [48,61,68]. Often, consumers are more likely to accept functional foods if they are familiar with the brand selling the product [48,61]. For example, Mirosa and Mangan-Walker [61] found that Chinese consumers most preferred to purchase functional foods from a foreign brand, followed by a well-known brand, and least preferred to purchase from a brand that was not familiar to them. Another study reported that consumers with knowledge about the leading brands tended to consume more functional foods [61]. However, Ares et al. [48] suggested that consumers who were more health conscious were inclined to consume functional foods that were not familiar to them.

#### 3.1.5. Health Information

The presentation of health information on functional food labels has been identified as a major determinant influencing consumer acceptance of functional foods [69]. Therefore, certain health information on food labels may improve consumers’ perceptions of health benefits and positively influence their acceptance [70]. Specifically, González-Díaz et al. [71] found that health information, such as the type of added functional ingredients and how they benefit human health, may lead to higher purchase intentions. Ahn et al. [72] explained that less informed consumers who did not fully understand the health attributes of functional products were unwilling to consume functional foods. Furthermore, Marette et al. [70] found that health information about the benefits of lowering cholesterol increased consumers’ purchase intentions for a fortified yogurt drink, and Markosyan et al. [73] found that potential health benefits information about antioxidants positively influenced consumers’ willingness to purchase functional foods. Additionally, Verneau et al. [32] found a positive relationship between providing information about the benefit of lycopene and consumers’ willingness to pay for lycopene-enriched products. Thus, providing accurate and objective health information about the efficacy of functional properties or attributes may increase consumers’ acceptance.

However, it should be noted that providing information about scientific uncertainty may reduce consumers’ willingness to purchase functional foods [70]. In addition, Ares et al. [33] explored the influence of using the functional ingredients’ name (common name vs. scientific name) on consumers’ healthiness perceptions and willingness to consume functional milk desserts. They found that using the common names (e.g., fiber, antioxidants) could increase consumers’ healthiness perceptions and their willingness to try functional food compared to using the scientific name (e.g., b-glucan, flavonoids).

A health claim—a common type of health information—has been described as a statement about the health benefits associated with consuming functional foods. Providing specific health claims may lead to increased product attractiveness, help consumers to link the health benefits with the effect, and eventually increase purchase intentions [27,33,48]. However, it has been reported that, in some cases, the format of health claims and their content may influence consumers’ preferences [20,35,74]. For instance, Van Kleef et al. [57] found that consumers preferred to consume functional foods when the health claim of the products involved reducing the risk of physiologically-based illnesses (reduction of cardiovascular disease and osteoporosis) when compared with psychologically-based health problems (reduction of stress and fatigue). Likewise, Siegrist et al. [75] found consumers were more inclined to purchase functional foods with physiological health claims (e.g., reduction of risk for cancer, reduction of risk for osteoporosis) compared with psychological health claims (e.g., reduction in lack of concentration, reduction of tiredness). Finally, Verbeke et al. [58] compared consumers’ intent to purchase functional foods with different types of health claims (e.g., nutrition claim, health claim, reduction of disease risk claim) and found that consumers had lower purchase intention for functional foods with a reduction of disease risk claim compared to those with nutrition and health claims.

### 3.2. Socio-Demographic Characteristics

Socio-demographic characteristics play a crucial role in consumers’ acceptance of functional foods [26,43,49,53,76,77,78,79]. The reviewed studies indicated the determinants included age, gender, educational level, household characteristics, geography and nationality, and marital status.

#### 3.2.1. Age

A number of studies have explored the influence of age on consumer acceptance. However, the findings were inconsistent. Several studies reported that older people were typically the primary consumers of functional foods (e.g., [32,60,75]). For example, de Jong et al. [80] found that people aged 65 years or older had a higher preference for many kinds of functional foods (e.g., yogurt with lactic acid bacteria). Some studies suggested that this was because they pay more attention to health issues than their younger counterparts [60,75]. However, other studies found younger people—aged 25 and below—were more interested in functional foods than their older counterparts (e.g., [81,82]). Carrillo et al. [83] attributed young peoples’ interest in consuming functional foods to their open-mindedness and willingness to try novelty foods. Similarly, other studies noted that young adults are an important future consumer group of functional foods. As examples, Carrillo et al. [83] found individuals between the ages of 18 and 34 to be potential functional food consumers and Markovina et al. [40] found individuals between the ages of 19 and 30 to be potential consumers in the future.

#### 3.2.2. Gender

Most studies reached a consensus about the influence of gender on functional foods acceptance. Specifically, they found that female consumers were more likely to consume functional foods than males [25,32,60,81]. A possible explanation for these results may be that women tend to have the primary role of purchasing and preparing foods for their family [59,79]. We found one study, conducted by Kljusuric et al. [49], which reported that female consumers from Coastal Croatia were not willing to pay increased prices for functional foods.

#### 3.2.3. Educational Level

Research has also found that educational level has a significant effect on consumer acceptance. Results from most studies indicated that educated people showed a greater intention to purchase functional foods [18,25,26,60,84,85,86]. For instance, Çakiroğlu and Uçar [81] found university graduates had a higher likelihood to consume functional foods, and de Jong et al. [80] concluded that, in general, education was associated with higher consumption of functional foods. Other researchers, however, observed that individuals with higher education levels tended to reject consuming functional foods, which could mean that people are not familiar with some functional foods’ health benefits, even though they have a higher level of education [29].

#### 3.2.4. Household Characteristics

Previous studies indicated that consumers’ household characteristics (e.g., income, household size) were relevant socio-demographic determinants that influence functional food acceptance. Results from the reviewed studies suggested that a higher income level was often positively associated with higher purchase intentions [18,62,82,87]. This could be explained in the sense that consumers with a higher income have the ability to spend more money on functional foods [83]. In terms of household characteristics, families with young children [63,77] or teenagers [58,88] were also more likely to purchase functional foods. Additional studies investigated the influence of household size on consumer acceptance [25,77]. For example, Markovina et al. [40] found that families with small household sizes were more willing to buy functional foods than those with larger household sizes. However, other studies found that an increased number of household members was positively associated with functional foods consumption [25,77].

#### 3.2.5. Nationality and Geographic Location

Consumers’ functional food acceptance can also be determined by geographical context and nationality [49]. For example, a study that examined geographical differences in consumers’ willingness to purchase functional foods found Croatian consumers from different geographical regions (e.g., interior versus the coastal areas) had different purchasing behaviors of functional foods [49]. In addition, Markosyan et al. [73] found consumers in Seattle, Washington, were less likely to pay a premium for functional products when compared to consumers in Spokane, Washington. Regarding the influence of consumers’ nationality, Bech-Larsen and Grunert [34] examined consumers’ attitude toward functional foods in the U.S., Denmark, and Finland. They found consumers in the U.S. and Denmark were less inclined to buy functional foods when compared to consumers in Finland [34]. Another study conducted by Labrecque et al. [89] found that French students who were skeptical about health information printed on functional food labels expressed less favorable attitudes toward functional foods when compared to French Canadian students. Furthermore, a comparison study of German and Chinese consumers found Chinese consumers had higher preferences for functional foods with health benefit claims than German consumers [27]. This could be attributed to skepticism among German consumers regarding the functional properties associated with the health benefit claims [27]. Given the traditional Chinese nutritional medicine culture, the idea that food may offer specific health benefits was much more prevalent in China, which could cause Chinese consumers to be more trusting of the health benefits (e.g., preventing certain diseases; [27]).

#### 3.2.6. Marital Status

Two of the reviewed studies discussed the influence of marital status on consumer acceptance. Bekoglu et al. [85] found consumers who were single were more likely to consume functional foods than married consumers, whereas Moro et al. [77] found consumers who were married or widowed were more willing to pay for functional foods than single or divorced consumers.

### 3.3. Psychological Characteristics

Psychological characteristics play a critical role in consumers’ decision-making processes toward functional food choices. This scoping review identified seven psychological factors that influence consumers’ acceptance of functional foods, including health consciousness, motivations, perceptions, beliefs, attitudes, trust and food neophobia, and nutrition knowledge.

#### 3.3.1. Health Consciousness

Health consciousness has been described as the degree to which individuals are aware of their health and tend to pursue health behaviors to maintain or improve their health status [90]. A positive relationship between heath consciousness and functional food purchase intention has been identified in previous studies (e.g., [45,47,66]). Specifically, the higher the level of health consciousness or concern consumers have, the stronger their intentions are to consume functional foods [45,47,55,66,91]. For example, consumers who cared more about their health status and diet tended to consume functional foods [36,92]. Similarly, consumers who expressed fear of cancer were more likely to purchase selenium enriched functional foods than those who were not frightened of cancer [93]. Kavoosi-Kalashami et al. [77] also found that consumers who had family members diagnosed with diabetes were inclined to pay higher prices for functional foods which included dietary sugar. Furthermore, Devcich et al. [42] found that individuals with higher levels of modern health worries (e.g., worrying about health risks from food additives, worrying about antibiotics in food) were willing to buy functional foods. Other studies have suggested that consumers who are concerned about their family members’ health status were interested in consuming functional foods [38,80]. For instance, Bui et al. [38] and Verbeke [80] found that having ill family member(s) or sick relative(s) may increase consumers’ functional food consumption.

#### 3.3.2. Motivations

Consumers’ health motivation has been identified as one of the most important internal motivations to consume functional foods. Health motivation is defined as “consumers’ goal-directed arousal to engage in preventive health behaviors” [94] (p. 210). Studies have found consumers health motivations (e.g., improving health, preventing the risk of certain diseases) determined their functional foods consuming intentions. The more health consciousness consumers were, the more they were motivated to consume functional foods [93,95]. For example, Chinese consumers who placed a greater importance on their mobility health—the ability of bones, joints, and muscles to function—were more willing to purchase functional foods to prevent mobility-related illnesses [61]. The more consumers considered eating healthy foods the greater their willingness was to purchase functional foods [61]. Similarly, Chang et al. [44] found that consumers who valued health, were health-oriented, and were interested in eating healthy food expressed higher purchase intentions toward functional beverage products.

We also identified additional internal motivations that may increase consumers’ acceptance of functional foods in the reviewed studies. For example, consumers who considered functional food as convenient (e.g., providing a “quick and easy” way to improve health), and those who believed that functional foods could ensure their standard of health tended to consume functional foods [25,96]. Several studies also revealed that consumers’ self-efficacy [93,97,98] and self-esteem [95] were important motivators for functional food consumption. Specifically, consumers could be motivated to consume functional foods if they felt confident in their ability to do so [93].

In addition to consumers’ internal motivations (e.g., health consciousness, health value, self-efficacy, self-esteem), several studies examined external social context factors that may stimulate consumers’ intention to consume functional foods. These factors, including social prestige, social norms, and subjective norms, represent social pressure or peer influence on purchase behavior. For example, Barauskaite et al. [99] found that the act of consumers purchasing products signals to their peers that they care about their personal health and well-being. Similarly, Nystrand and Olsen [97] found that social pressure (descriptive and injunctive norms) was a strong predictor of Norwegian consumers’ intent to purchase functional foods. In addition, Nguyen et al. [100] found that subjective norm was positively correlated with consumers’ intention to purchase functional foods in Vietnam, which aligns with results from Rezai et al. [101] who found that subjective norms had a positive effect on consumers’ acceptance of functional foods. Furthermore, Phuong and Dat [102] asserted that the higher social prestige, the higher consumers intent was to purchase functional foods. Barauskaite et al. [99] also investigated social motivations behind the consumption of functional foods and found that consumers’ tendency for conspicuous consumption was positively associated with self-reported purchase rate of functional foods.

#### 3.3.3. Perceptions

Consumers may consume functional food if such food is perceived as healthy [34,88]. For example, Rezai et al. [101] found that consumers who perceived greater benefits from functional foods (e.g., reducing the risk of health problems, improving skin conditions, providing daily nutrition) were more accepting of functional foods. Another study found that consumers’ purchase intentions toward functional foods increased if they perceived the healthfulness of the products to be personally relevant to their health status [41]. Likewise, Jahn et al. [46] tested a conceptual model of consumers’ purchase intention toward Vitamin D fortified food and found their perceived personal benefit of consuming Vitamin D functional foods influenced their acceptance of such products. Finally, Xin and Seo’s [103] study revealed that consumers’ intention to purchase Korean functional foods was influenced by their perceived behavioral control. Specifically, if consumers perceived it to be easy for them to purchase functional foods (e.g., having time to buy functional foods, knowing where to by functional foods), then they tended to accept functional foods.

#### 3.3.4. Beliefs

Beliefs are another psychological determinant that may affect consumer acceptance of functional foods. In general, the more health benefits consumers believe functional foods offer, the more likely they are to accept functional foods [38,39,89]. Previous studies have demonstrated that consumers who believed functional foods could improve their well-being and quality of life were inclined to accept functional foods [86,98]. For example, Vecchio et al. [98] found consumers were more willing to purchase omega-3 enriched mozzarella if they believed health benefits included preventing cardiovascular and rheumatic diseases. Results from other studies indicated that consumers who believed in the value and benefit of functional foods for personal health were more likely to accept them [31,80]. For example, Corso et al. [87] found consumers were inclined to accept antioxidant-enriched soluble coffee if they believed health benefits of the product included obtaining the recommended daily intake of certain components or helping them take control of their health.

#### 3.3.5. Attitudes

Attitudes typically predict behavior [104]. Many studies have found that consumers’ attitudes guided their overall evaluation of possible consequences of consuming functional foods [37,46,96]. Consumers who have positive attitudes toward functional foods were more willing to consume functional foods than those whose attitudes were not positive [55,102,105]. On a related note, Kavoosi-Kalashami et al. [77] found consumers’ healthy purchase attitudes and their attitudes toward health benefits of consuming dietary sugar had a positive significant effect on their willingness to pay for dietary sugar functional foods. Finally, Szakály et al. [18] suggested that consumers who had more positive attitudes toward functional foods were more willing to pay a premium for the products if they believed the functional foods had health benefits.

Previous studies have identified many factors that influence consumers’ attitudes toward functional foods [40,42,100,106]. For example, Chen [106] found consumers who were more health conscious had a more positive attitude toward functional foods and were more willing to consume such foods. Other studies conducted more recently found similar results indicating health consciousness influences consumers’ attitudes toward functional foods [45,100]. Likewise, Devcich et al. [42] and Chen [106] stated that people who had modern health worries expressed a more positive attitude toward functional foods, which increased their willingness to consume them. Markovina et al. [40] also identified a variety of factors that influenced young Croatia consumers’ attitudes toward functional foods—health awareness and confidence, lack of trust for functional foods, and perceived price and quality ratio. Nguyen et al. [100] similarly reported that perceived price influenced consumers’ attitudes toward functional foods. Specifically, they found perceived price of functional yogurt had a negative impact. Finally, Jung et al. [47] found that perceived taste was positively correlated with U.S. consumers’ attitudes toward functional antioxidant-enriched foods.

Attitude is a multifaceted concept that consists of hedonic and utilitarian dimensions [107,108]. The hedonic attributes, or values of functional foods pertaining to taste pleasures or enjoyments, provide sensation experiences for consumers, and the utilitarian values, or benefits of functional food, offer health-related benefits [97,109]. Nystrand and Olsen [97] found Norwegian consumers’ attitudes toward consuming functional foods were positively influenced by utilitarian values and negatively influenced by hedonic values.

Urala and Lähteenmäki [7] developed a scale to evaluate consumers’ attitude toward functional foods to better predict their acceptance. The scale contains four distinct dimensions: reward from consuming functional foods, necessity for functional foods, confidence in functional foods, and safety of functional foods. It was used in several of the reviewed studies to evaluate consumers’ attitudes toward functional foods [39,83,85,92]. These studies concluded that consumers who perceived more reward from consuming functional foods, believed functional foods were necessary, were confident in functional foods, and perceived higher safety of functional foods had a positive attitude toward functional foods and were more willing to consume them [39,83,85,92].

#### 3.3.6. Trust and Food Neophobia

Functional food is a type of novel food that does not have a long history of consumption. The process of producing functional food (e.g., adding new or unusual ingredients) uses food technology that is relatively unfamiliar to consumers. The novel, unfamiliar technology may cause consumers to be skeptical or reluctant to adopt some functional foods [17,110]. In addition, functional foods are designed to improve health conditions or reduce the risk of health problems, but it is difficult for consumers to verify concrete and tangible health effects at the point of consumption. Therefore, the degree of consumers’ trust, a complex psychological factor, may influence consumer acceptance of functional foods [61].

Consumers tend to accept functional foods if the perceived health benefits outweigh the perceived risk. For example, Huang et al. [45] found that Chinese consumers who trusted entities involved in the food system (e.g., governments, food manufacturers, food retailers) tended to purchase functional foods. Additionally, the degree of trust in food science [32], the food industry [76], and food safety control systems [36] affected consumers’ willingness to purchase functional foods. Results from Shan et al.’s [111] study indicated that consumers were skeptical about the health effects from processed functional meat products.

In addition, the importance of trust in advertising was highlighted in several studies [54,91,112]. For instance, consumers preferred to purchase functional foods if they received health information from channels perceived as credible [54]. Sandmann et al. [91] found that consumers perceived professional health care organizations (e.g., physicians and health insurance companies) to be some of the most credible sources of information. Another study conducted by Melbye et al. [112] found that the physical features of an endorser (a person used in an advertising) on a functional energy drink influenced consumers’ assessment of the health-related benefits. Specifically, if advertising was communicated by a person with a lean figure (e.g., sportier person), consumers considered the health benefits to be more credible. In addition, Chinese consumers tended to trust information advertised or publicized through authoritative figures, including published scientists and political leaders [61].

Food neophobia has been used to predict consumers’ tendency to avoid the use of novel foods [89,113,114] as food-neophobic individuals are skeptical and hesitant to try novel foods [115]. In terms of the influence of food neophobia on consumers’ acceptance of functional foods, the reviewed studies suggested that, to a certain degree, consumers demonstrated a food-neophobic attitude toward adopting functional foods. For example, studies conducted in Europe found that food neophobia had a direct negative effect on consumers’ attitude toward adopting functional foods [32,116], but others argued that the effect was indirect. Huang et al. [45], for example, found that food neophobia moderates the relationship between Chinese consumers’ trust of food systems and purchase attitude toward functional foods. In addition, Moons et al.’s [66] study conducted in Belgium found that food neophobia only negatively influenced foodies’ intent to adopt functional foods. Foodies refer to people interested in novel food and its health- and/or environmental-related benefits [66]. However, food neophobia did not influence the intent of sporting individuals, or individuals who “are interested in the positive effects of food consumption on their health, physical performances and body shape” [66] (p. 155). Food neophobia also had no effect on vegetarians, or individuals who “abstain from the consumption of meat, and eventually from by-products of animal slaughter” [66] (p. 155). Siegrist et al. [27] further found that food neophobia had a negative effect on Chinese consumers’ willingness to buy functional foods, but it did not affect German consumers’ willingness to purchase such foods.

#### 3.3.7. Nutrition Knowledge

Nutrition knowledge is defined as “a scientific construct that nutrition educators have created to represent individual’s cognitive processes related to information about food and nutrition” [117] (p. 239). Adequate nutrition knowledge could change dietary attitudes and habits, and ultimately influence their acceptance [89,118]. According to a review about consumer motivations and expectations about functional foods, nutritional knowledge severs as the most important factor influencing consumer acceptance of functional foods [119]. In addition, Stojanovic et al. [63] found that higher levels of knowledge about health information positively affected consumers’ frequency of consuming functional foods. Similarly, La Barbera et al. [116] found that consumers with higher levels of knowledge about functional foods tended to pay higher premium prices for functional foods than those with lower levels of knowledge. Several other studies also confirmed the positive effect of knowledge on consumers’ functional foods acceptance [25,78,80,103]. A study conducted by Verneau et al. in 2019 [32] identified knowledge as a moderator between information shock and willingness to purchase functional foods. Specifically, people with less knowledge about functional foods increased their likelihood of buying functional foods after they received information about their health benefits. Similarly, Lu [56] found that consumers’ level of knowledge moderated their perception of the carrier–ingredient fit, or level of perceived ‘naturalness’ of the carrier–ingredient combination, and thus their purchase intentions. Conversely, Verbeke [80] found that consumers’ level of knowledge had a negative effect on their acceptance of functional foods.

### 3.4. Behavioral Characteristics

Consumers’ behavioral characteristics have been regularly used to investigate how consumers choose healthy foods. Previous studies demonstrated that individuals who were health conscious tended to engage in health-related behaviors (e.g., adopting a healthy lifestyle; [120,121]). For example, several studies found consumers who had a heathier lifestyle (e.g., consuming natural foods, maintaining life equilibrium, and exercising [77,92,106,121]; and who tended to engage in health-related behaviors (e.g., being physically active [43], taking nutraceuticals or dietary supplements [66,86], or demonstrating concern for their body image ([61] and Moro et al. [77])) tended to accept functional foods compared to those who did not. Additionally, de Jong et al. [80] compared lifestyles between Dutch functional food consumers and non-consumers and found a relationship between moderate or high vegetable intake and functional food consumption. In addition, de Jong et al. [80] found smokers were more likely to consume cholesterol-lowing margarines, and Rezai et al. [82] found that consumers who subscribed to cooking or health magazines, were vegetarians, and who had experience in a food production company were more aware of functional foods. Peng et al. [88] also found consumers who previously consumed calcium- or vitamin-enriched milk or orange juice were more likely to accept conjugated linoleic acid-enriched dairy products. Finally, Bekoglu et al. [85] and Carrillo et al. [83] found that consumers who were more innovative, meaning they tended to seek novelty products, were interested in consuming functional foods.

### 3.5. Physical Characteristics

Physical characteristics pertain to related to individuals’ body condition or feature resulting from physical development. It is well known anecdotally that, if people have experienced physical health issues, they will most likely be more concerned about consuming healthy foods. This concept has also been tested empirically in the functional food domain. For example, Landström et al. [86] found that Swedish consumers who had a diet-related problem (e.g., high blood pressure, high cholesterol, or diabetes) were interested in consuming cholesterol-lowering functional foods. Likewise, Chen [106] found consumers who reported more subjective health complaints (e.g., flu, musculoskeletal pain, pseudoneurology, gastrointestinal problems, allergies, etc.) had positive attitudes toward functional foods and were inclined to consume such foods. In addition, Brečić et al. [25] found a positive relationship between consumers’ self-reported body mass index and their functional foods consumption. This could be because consumers with a higher body mass index may be seeking to change their unhealthy lifestyle and, therefore, consume more functional foods [25,84]. Another study conducted by de Jong et al. [80] revealed that consumers with poor subjective health were inclined to use cholesterol-lowering margarine.

## 4. Discussion

Our scoping review identified a wide range of determinants and sub-determinants affecting consumer responses to functional foods. It should be noted that the extent to which determinants influence consumer acceptance may interact with other determinants dynamically. As mentioned earlier, findings from previous studies were not consistent, nor were the relationships among these determinants always consistent or significant. Thus, it is difficult to establish the existence of direct or linear relationships among these determinants. Instead, there are a number of determinants that influence consumer acceptance collectively. The complexity of the determinants and inconsistency of findings proposed challenges for drawing generalized conclusions about the extent and accurate direction of the variables’ influence on consumer behavior. This scoping review attempted to describe general trends as they relate to consumers’ acceptance of functional foods by synthesizing some of the most robust and comprehensive findings that have been reported in the literature.

Before launching novel products, it is important to explore the influence of product characteristics on consumer preferences. Scientists and functional food manufacturers should carefully consider how to choose functional carrier ingredients and set pricing. Because functional foods improve consumer health and well-being, and because consumers purchase functional foods with this in mind, they tend to be more accepting of healthier carriers (e.g., yogurt; [34,57,58]. Perhaps consumers perceive that the use of less healthy carriers (e.g., ice cream, sausage, etc.) counteracts or diminishes the effect of the functional ingredient on their health. Still, additional research is needed to investigate consumer responses to functional foods that use less healthy carriers. It is possible that these products could appeal to subpopulations of consumers who tend to eat less healthily and who have not yet accepted functional foods as a part of their diet. In addition, sensory studies on product taste should be conducted early in the research and development process, as our findings revealed that as sensory preferences are likely the critical driver influencing functional food consumption. Consumers are also more likely to pay a premium to purchase functional foods associated with improvements in household health, particularly in differing phases of human development and post-disease diagnoses for members of their household.

Communications and marketing professionals in the industry should carefully consider how to design labels, position brands, and develop advertising campaigns. According to results from the reviewed studies, functional food product labels should accurately and precisely communicate information about the type of functional ingredient and its benefits to human health [32,70,71,73]. Providing this type of health information on labels can significantly increase consumers’ acceptance. It is important that future research seek to determine the situations and contexts in which certain health claims are more effective at increasing consumer acceptance than others. For example, physiological health claims and psychological health claims have prompted various consumer responses. Thus, revealing additional evidence to inform when, where, and for whom certain health information is most appropriate will ensure that communications and marketing professionals in the industry design effective product labels and deliver other compelling promotional materials.

In addition, because branding can influence consumer acceptance of functional foods [48,61], it is important that communications and marketing professionals working for functional food companies prioritize positioning the company’s brand to create brand associations in the minds of consumers who constitute their target audience. As a result, their target audience should be able to distinguish how the brand differs from competitors. More effective branding could increase consumers’ familiarity, thereby increasing their acceptance [48,61]. This can be accomplished by implementing a brand positioning strategy after comprehensively understanding the functional foods market, which this scoping review can help professionals in the industry achieve.

Another aspect of marketing that influences consumer acceptance of functional food is advertising, especially as it relates to trust and credibility [54,91,112]. Marketing professionals working for functional food companies should consider using health care professionals [91], scientists [61], or healthy-looking individuals who appear to be exercise conscious [112] in functional food advertising campaigns and associated promotional materials. Because consumers perceive individuals in these roles as credible and trustworthy, functional food companies should rely on them to share the company’s message.

It should be noted that previous studies investigating the effect of socio-demographic characteristics on consumers’ functional food acceptance were inconsistent in their findings. As a result, it is difficult to generalize the demographic characteristics of functional food consumers legitimately, since the various studies reviewed implemented different types of functional products, methods, and populations. Nevertheless, different demographic groups may prefer different types of functional foods [26]. In addition, consumers’ food choices may be influenced by their nationality. Thus, researchers in the functional food domain should note that findings from one geographic area or cultural group may not be applicable to other geographic or cultural contexts. Therefore, when releasing functional foods to the consumer market, socio-demographic characteristics should be carefully considered to target specific consumer groups.

Furthermore, our findings suggest that the relationship between psychological determinants and consumer acceptance of functional foods is important, but complex. The psychological determinants are interdependent and correlated. There is a conscious or unconscious psychological response when consumers purchase new functional food products. Among the psychological determinants, consumers’ health consciousness exerts a positive impact on consumer acceptance. In addition, a positive relationship between consumers’ perceptions regarding the health benefits of functional foods and their acceptance were identified in previous studies [41,101]. Likewise, consumers who believe the health benefits of consuming functional food are likely to use functional foods. Furthermore, consumers’ attitudes are influenced by many factors and vary based on consumers’ cultural context. Thus, understanding the sub-determinants collectively could help consumers develop positive attitudes toward using functional foods and, ultimately, increase their acceptance. For example, emphasizing the rewards and the necessity of using functional foods may cultivate consumers’ positive attitudes toward functional foods [92].

Regarding trust and food neophobia, as functional foods are produced by adding or changing healthful ingredients, consumers may perceive consuming such foods as a possible risk due to being less familiar with the technology and potential unknown consequences of consuming food produced using the technology [17,110]. However, consumers tend to accept functional foods if the perceived health benefits outweigh the perceived risk. Therefore, consumers’ suspicion and distrust could be alleviated if they become more familiar with the health benefits that functional foods provide. In addition to providing this information on product labels, as previously noted, further efforts should be made to inform and educate consumers about health benefits that result from consuming functional foods. It can be assumed that consumers are more likely to purchase functional foods if appropriate information about the health benefits is provided [32,70,71,72,73].

Similarly, because consumers’ knowledge pertaining to functional foods can influence their perceptions and purchase behavior toward such products, educating consumers to increase their knowledge about the health benefits of consuming functional foods could also be an effective way to improve their health awareness and consciousness. Once consumers have common knowledge about how to evaluate health benefits from functional foods, their acceptance may increase [122,123]. In addition, functional food industry experts should inform consumers about functional food processing and production technology. Doing so could alleviate consumers’ concerns about adverse side effects and increase their trust and confidence in functional food production institutions and food technologies.

The behavioral characteristics we identified in the reviewed studies may help scientists and other functional food experts understand why consumers prefer to choose functional foods rather than conventional foods. Generally, consumers who adopt a healthy lifestyle or engage in health-promoting behaviors tend to accept functional foods to maintain their well-being. Additionally, consumers who previously consumed functional foods and consumers who seek novelty products tend to accept functional foods. However, these behavioral characteristics are influenced by conscious and unconscious motives [121]. Therefore, based on the limited number of studies we included in our review, it is difficult to confirm an association between consumers’ behavioral characteristics and their acceptance of functional foods. Still, these findings can provide insight into the consumer groups that communications and marketing professionals in the industry should considering targeting when launching new functional foods or promoting existing functional foods.

Finally, consumers with certain physical characteristics (e.g., diet-related health problems, subjective health complaints, higher body mass index, or poor subjective health) are inclined to consume functional foods. In the studies we reviewed, we found evidence to suggest consumers who have certain physical characteristics accept functional foods, likely because they seek healthy foods that can improve their health status. Therefore, our previous recommendation to emphasize the health benefits of consuming functional foods, specifically how they can help improve consumers’ physical ailments, could be an effective communications strategy to increase consumers acceptance of functional foods.

### Limitations and Future Recommendations

This scoping review was conducted to identify and synthesize prominent determinants that influence consumers’ acceptance of functional foods and attempts to integrate the contradictory and inconsistent research findings. However, some limitations need to be acknowledged and can be addressed in future research. First, studies included in the scoping review were limited to four databases. Studies that investigate consumers’ acceptance of functional foods might exist in other databases. Therefore, other factors related to consumer acceptance may exist that are not identified in this review. Future research should build upon this scoping review by using additional databases to search for other determinants that influence consumer acceptance of functional foods.

Second, an important limitation is the heterogeneity across studies included in this review. The studies included used a variety of instruments and scales, methodologies, types of functional foods, and outcome measurements. The heterogeneity of methods and measurements applied in the reviewed studies limited us to perform a quality assessment for the included studies. Therefore, it is possible that if future researchers conduct a similar review and only include studies that use standardized measurement or a consistent research design, they may be able to conduct a quality assessment. However, a quality assessment is not mandatory for scoping reviews [23]. In addition, we recommend researchers conduct systematic reviews and meta-analyses in the future to investigate important determinants influencing consumer acceptance.

Third, the review attempted to synthesize the determinants that have causal relationships with consumers’ acceptance through observable and numerical measurements. Thus, only quantitative studies were included and analyzed. Additionally, the studies included focused on modified or altered functional foods instead of whole functional foods, which helps to create a clear distinction from conventional foods. Additionally, all populations investigated in the reviewed studies were 18 years and older, so participants were food purchasers and contributed to the public health perspective. Studies that focused on a specific population (e.g., women, older people, children, comparisons between children and adults) were not included in the scoping review because we sought to provide information that can be generalized well to the general population as opposed to specific populations. Finally, the studies included in this scoping review were limited to those published in English and in peer-reviewed journals. In summary, findings described herein can enable those working with functional foods to better predict public acceptance toward different types of functional products in varied contexts. They can also provide key insights to develop effective communication strategies that may ultimately increase public acceptance of functional foods and improve the health of many consumer groups.

## 5. Conclusions

Given the competitive nature of the functional foods market and consumers’ complex process of accepting novel foods, an understanding of the determinants that influence consumer acceptance and their relationships is key to a successful product launch and development of marketing strategies for the novel functional foods industry. Based on a review of 75 studies conducted around the globe that empirically investigated consumers’ acceptance of functional foods, our scoping review identified a wide range of determinants, and we classified the determinants into five categories, which are product characteristics, socio-demographic characteristics, psychological characteristics, behavioral characteristics, and physical characteristics. We attempted to provide insights for leaders and scientists in product development, and for communications and marketing specialists in the industry who serve as the liaison between functional foods and the public. Therefore, these five categories of prominent determinants should be considered and used to inform the research of scholars working in the functional food domain and, ultimately, to inform the successful launch of novel functional foods.

## Figures and Tables

**Table 1:** Inclusion and exclusion criteria for article screening.

**Inclusion Criteria**
1. Quantitative studies examining the possible determinants related to consumer behaviors toward functional food.
2. Studies focusing on modified or altered functional foods.
3. Participants restricted to 18 years and older.
4. Studies published in English and in peer-reviewed journals between January 2000 and October 2020.
**Exclusion Criteria**
1. Qualitative studies.
2. Studies investigating functional foods that were not altered or modified.
3. Studies that focused on a specific population (e.g., women, older people, children, and comparisons between children and adults).
4. Book chapters, secondary articles, and reviews.
5. Studies published in a non-English language and before January 2000 and after October 2020.
6. Studies focusing on production side of functional foods (e.g., the development process, evaluation of functional ingredients, and packing methods).
7. Studies comparing consumer acceptance between conventional food and functional food or organic food and functional food.

## Data Availability

Not applicable.

## Appendix A

**Table A1:** Characteristics and key findings of included studies.

Authors	Year	Research Method	Functional Foods	Continent	Sample Size	Key Findings	Categories (Determinants)	Outcomes
Ares et al. [33]	2009	Conjoint study	Functional milk desserts’ images	Europe	82	(a) Providing health claims was necessary for consumers to link health benefits to functional foods’ effect on their health, and eventually increase their purchase intentions; Compared to using scientific names (b-glucan or flavonoids), the use of common names (fiber or antioxidants) could increase consumers’ health perceptions and their willingness to try functional food. (b) Older people and females were more willing to try functional foods.	(a) Product characteristics (health information). (b) Socio-demographic characteristics (age, gender)	Willingness to try
Ahn et al. [72]	2016	Choice experiment	Red ginseng concentrates	Asia	240	(a) Not fully understanding health attributes of functional foods was one of the major barriers for consuming functional foods.	(a) Product characteristics (health information)	Preferences and willingness to pay
Ares et al. [53]	2007	Survey	Functional food concept	Europe	200	(a) Consumers were more likely to accept functional foods if they perceived the carrier to be healthy. (b) Different socio-demographic (age, gender) groups had different preferences toward functional foods.	(a) Product characteristics (carrier/ingredient combination). (b) Socio-demographic characteristics (age, gender)	Willingness to try
Ares et al. [48]	2010	Conjoint study	Yogurts enriched with antioxidants and fiber	Europe	103	(a) Price had a significantly negative effect on consumers’ consumption of functional yogurts. (b) Consumers were more likely to accept functional foods if the brand was familiar to them.	(a) Product characteristics (price). (b) Product characteristics(brand)	Functional foods choice
Barauskaite et al. [99]	2018	Survey	18 functional products	Europe	900	(a) Conspicuous consumption was positively associated with functional foods purchase rate.	(a) Psychological characteristics (motivation)	Purchase rate of functional foods
Barreiro-Hurlé et al. [36]	2008	Choice experiment	Resveratrol-enriched red wine	Europe	300	(a) The more consumers cared about their health and a healthy diet, the more likely they were to buy functional foods. (b) Consumers who trusted food technology development and food safety control were more willing to buy functional foods.	(a) Psychological characteristics (health consciousness). (b) Psychological characteristics (trust in food technology)	Willingness to buy
Bech-Larsen & Grunert [34]	2003	Conjoint study	24 standard full-profile stimuli	Europe	1553	(a) Denmark and U.S. consumers were less inclined to accept functional foods compared to Finnish consumers. (b) Consumers considered inherently wholesome foods (e.g., orange juice, yogurt) as being healthier carriers than unwholesome foods (e.g., spreads).	(a) Socio-demographic characteristics (nationality). (b) Product characteristics (carrier/ingredient combination)	Functional foods perceptions
Bechtold & Abdulai [37]	2014	Choice experiment	Functional dairy product	Europe	1309	(a) Consumers with different attitudes (skeptics, advocates, and neutrals) had different preferences toward functional food attributes.	(a) Psychological characteristics (attitude)	Willingness to pay
Bekoglu et al. [85]	2016	Survey	Concepts about different types of functional foods	Asia	695	(a) Consumers with a higher educational level and who were single were more likely to use functional foods. (b) Consumers’ attitudes toward the necessity of functional foods positively influenced their functional food consumption. (c) Innovative consumers who had the tendency to seek novelty products were likely to consume functional foods.	(a) Socio-demographic characteristics (education, marital status). (b) Psychological characteristics (attitude). (c) Behavioral characteristics (seeking innovativeness)	Functional food consumption
Bimbo et al. [68]	2018	Choice experiment	Functional probiotic yogurts	Europe	229	(a) There was a negative correlation between consumers’ body image dissatisfaction and the number of functional yogurts they purchased. (b) Consumers who had more knowledge regarding functional yogurt brands purchased more functional foods.	(a) Physical characteristics (body mass index). (b) Product characteristics (brand)	Functional food purchased
Brečić et al. [25]	2014	Survey	Functional food concept	Europe	424	(a) Female consumers, older consumers, and consumers with higher levels of education were likely to consume functional foods; Consumers with a larger family were willing to consume functional foods. (b) Consumers who believed functional foods were healthy and convenient were willing to consume them. (c) There was a positive correlation between consumers’ self-reported body mass index and their functional food consumption. (d) Nutrition knowledge positively influenced consumers’ functional food acceptance.	(a) Socio-demographic characteristics (gender, age, education, household size). (b) Psychological characteristics (health motivations). (c) Physical characteristics (body mass index). (d) Psychological characteristics (nutrition knowledge)	Functional food consumption
Bruschi et al. [30]	2015	Experimental auction	Anthocyanin-containing bakery	Europe	207	(a) Young Russian consumers were concerned about the naturalness and health properties of functional foods. (b) Taste was the most important attribute of functional foods.	(a) Product characteristics (carrier/ingredient combination). (b) Product characteristics (taste)	Willingness to pay
Bui et al. [38]	2015	Survey	Functional food concept	Asia	217	(a) Consumers’ high level of acceptance was associated with more perceived benefits from functional foods. (b) The presence of an ill family member may increase consumers’ functional food consumption.	(a) Psychological characteristics (beliefs). (b) Psychological characteristics (health consciousness)	Consumer acceptance
Büyükkaragöz et al. [60]	2014	Survey	12 functional food items	Asia	808	(a) Female consumers, older consumers, and well-educated consumers are more likely to consume functional foods. (b) Price influences consumers’ functional food consumption. (c) Consumers who took vitamin supplements were likely to accept functional foods.	(a) Socio-demographic characteristics (gender, age, educational level). (b) Price. (c) Behavioral characteristics (health related behavior)	Functional food consumption
Çakiroğlu & Uçar [81]	2018	Survey	Functional milk and dairy products; cereal product; beverages; other functional products	Asia	1182	(a) Consumers between the ages of 18 and 25, female consumers, and university graduates were likely to purchase functional foods.	(a) Socio-demographic characteristics (age, gender, educational level)	Purchase intention
Carrillo et al. [83]	2013	Survey	Functional food concept	Europe	197	(a) Consumers between the ages of 18 and 35 tended to consume more functional foods; Female consumers were more interested in functional foods. (b) Consumers’ positive attitudes (reward, necessity, confidence) and novelty positively influenced their functional food consumption. (c) Healthiness and natural content were motives for consumers to consume functional foods.	(a) Socio-demographic characteristics (age, gender). (b) Psychological characteristics (attitude). (c) Psychological characteristics (motivations)	Functional food consumption
Chang et al. [44]	2020	Survey	Functional beverages	Asia	213	(a) Consumers who were health-oriented, valued health, and interested in eating healthy food had higher purchase intentions for functional beverages products.	(a) Psychological characteristics (motivations)	Purchase intention
Chen [92]	2011a	Survey	Eight functional foods	Asia	533	(a) Consumers who had a positive attitude toward functional foods were willing to buy functional foods. (b) Health consciousness had a positive influence on consumers’ functional food preferences. (c) Healthy lifestyle positively influenced consumers’ functional foods preferences.	(a) Psychological characteristics (attitude). (b) Psychological characteristics (health consciousness). (c) Behavioral characteristics (lifestyle)	Willingness to use
Chen [106]	2011b	Survey	Eight functional foods	Asia	633	(a) Consumers who were health consciousness had more positive attitudes toward functional foods and were willing to use functional foods; Consumers who had modern health worries had more positive attitudes toward functional foods and were willing to consume functional foods. (b) Consumers who lived a healthier lifestyle were willing to consume functional foods; (c) Consumers who reported more subjective health complaints had more positive attitudes toward functional foods and were willingness to use them.	(a) Psychological characteristics (attitude, health consciousness). (b) Behavioral characteristics (lifestyle). (c) Physical characteristics (subjective health complaints)	Willingness to use
Corso et al. [87]	2018	Survey	Soluble coffee enriched with antioxidants	South America	270	(a) Older consumers who had a higher educational level and a higher income were more likely to accept functional foods. (b) Consumers who believed in the health benefits were more inclined to accept antioxidant-enriched soluble coffee. (c) Consumers’ knowledge positively influenced their functional food acceptance.	(a) Socio-demographic characteristics (age, educational level, income). (b) Psychological characteristics (beliefs). (c) Psychological characteristics (knowledge)	Consumer acceptance
Cox & Bastiaans [93]	2007	Survey	Se-enriched foods	Asia	200	(a) Consumers who feared cancer were willing to purchase selenium-enriched foods. (b) Consumers’ self-efficacy was an important motivator for consuming functional foods.	(a) Psychological characteristics (health consciousness). (b) Psychological characteristics (motivation)	Likelihood to purchase
de Jong et al. [80]	2003	Survey	Functional food concept	Europe	1552	(a) Female consumers, older consumers, and consumers with higher levels of education had a higher preference for functional foods. (b) There was a correlation between consumers’ moderate or high vegetable intake and functional food consumption; Smokers were more likely to consume cholesterol-lowing margarines. (c) Consumers with poor subjective health were more inclined to use cholesterol-lowering margarine.	(a) Socio-demographic characteristics (gender, age, educational level). (b) Behavioral characteristics(lifestyle). (c) Physical characteristics (poor subjective health)	Use of functional foods
Dean et al. [41]	2012	Survey	Foods with health-related claims	Europe	2385	(a) Consumers’ purchase intentions toward functional foods increased if their perceived healthiness of the products were personally relevant to their health status.	(a) Psychological characteristics (perceptions)	Functional food perceptions
Devcich et al. [42]	2007	Survey	Synthetic additives in margarine and yoghurt	Oceania	390	(a) Consumers having modern health worries expressed a more positive attitude toward functional foods and included to consume functional foods.	(a) Psychological characteristics (health consciousness)	Functional food consumption
Huang et al. [45]	2019	Survey	Functional food concept	Asia	1144	(a) Price negatively affected consumers’ purchase intentions, but this negative effect could be intervened by health consciousness. (b) Consumers who were more health conscious expressed more positive attitudes toward functional foods and were likely to purchase them. (c) Chinese consumers who trusted the food system were likely to purchase functional foods.	(a) Product characteristics (price). (b) Psychological characteristics (health consciousness). (c) Psychological characteristics (trust)	Purchase intention
Huang et al. [54]	2020	Survey	Yogurt, non-alcoholic beverage, and biscuits	Asia	1144	(a) The functional foods carrier influenced consumers’ perceptions and purchase intentions. (b) Consumers’ perceived trust in mass media influenced their purchase intentions; Consumers preferred to purchase functional foods if they received health information from credible channel.	(a) Product characteristics (carrier/ingredient combination). (b) Psychological characteristics (trust)	Perceived attractiveness and purchase intention
Jahn et al. [46]	2019	Survey	Vitamin D-fortified food	Europe	1263	(a) Positive attitudes toward functional foods, population nutrient deficiency awareness, and perceived appropriateness of fortified-products influenced consumers’ decisions to purchase Vitamin D-fortified foods.	(a) Psychological characteristics (attitude). (b) Psychological characteristics (health consciousness). (c) Product characteristics (carrier/ingredient combination)	Purchase intention
Jeżewska-Zychowicz & Królak [96]	2015	Survey	Cereal fortified with fiber	Europe	1000	(a) Consumers who placed a high-level of importance on food quality as a guarantee of health were willing to consume fiber-enriched functional foods. (b) Consumers who had positive attitudes toward food technologies were willing to consume functional foods.	(a) Psychological characteristics (motivation). (b) Psychological characteristics (attitude)	Consumption intentions
Jezewska-Zychowicz [39]	2009	Survey	Cholesterol-lowering spreads, probiotic yoghurt, juice with added calcium, low-fat mayonnaise, and energetic beverages	Europe	275	(a) Consumers’ beliefs in functional foods health benefits positively influenced their acceptance. (b) Consumers’ attitude positively influenced their willingness to buy.	(a) Psychological characteristics (beliefs). (b) Psychological characteristics (attitude)	Willingness to buy
Jung et al. [47]	2020	Survey	Antioxidant-infused sugar-free chewing gum	North America	368	(a) Perceived taste was positively correlated with U.S. consumers’ attitudes toward functional foods. (b) Consumers who were more health consciousness had more positive attitudes toward functional foods and were more likely to purchase them.	(a) Product characteristics (taste). (b) Psychological characteristics (health consciousness). (c) Psychological characteristics (attitude)	Intention to purchase
Kavoosi-Kalashami et al. [76]	2017	Contingent valuation	Dietary sugar	Asia	125	(a) Consumers’ age, educational level, family size, and income affected their willingness to pay for functional foods. (b) Consumers who had a record of diabetes in their family were willing to pay for functional foods with dietary sugar. (c) Consumers’ attitudes toward health benefits had a significant direct effect on their willingness to pay for functional foods with dietary sugar.	(a) Socio-demographic characteristics (age, educational level, family size, income). (b) Psychological characteristics (health consciousness). (c) Psychological characteristics (attitude)	Willingness to pay
Kljusuric, et al. [49]	2015	Survey	Functional food concept	Europe	687	(a) Consumers’ age, gender, educational level, income, and geographic location affected their functional foods consumption.	(a) Socio-demographic characteristics (age, gender, educational level, income, geography)	Functional foods choice
Kraus et al. [26]	2017	Survey	Functional food concept	Europe	200	(a) Consumer groups that differ by gender and age had different preferences for functional foods carriers. Female consumers and older male consumers preferred cereal products as functional foods carriers, whereas young males preferred meat products as functional foods carriers; Female consumers were more health-conscious toward functional food carriers and they were quality-oriented, whereas young male consumers were less health-consciousness toward functional foods carriers; Consumers with a university education were more interested in functional foods.	(a) Socio-demographic characteristics (age, gender, education)	Purchase intention
Kraus [95]	2015	Survey	Functional food concept	Europe	200	(a) Consumers who were more motived to improve their health and prevent the risk of becoming less healthy were inclined to consume functional foods.	(a) Psychological characteristics (motivation)	Functional foods consumption
Krutulyte et al. [55]	2011	Survey	Seven different functional foods categories	Europe	999	(a) Consumers preferred to purchase functional food product combinations that were more familiar to them. (b) Consumers who were more concerned about their health had a higher intention to purchase functional foods. (c) Consumers who had positive attitudes toward functional foods were more willing to purchase them.	(a) Product characteristics (carrier/ingredient combination). (b) Psychological characteristics (health consciousness). (c) Psychological characteristics (attitude)	Purchase intention
La Barbera et al. [116]	2016	Experimental auction	A crushed tomato enriched with lycopene	Europe	100	(a) Consumers with a higher level of knowledge about lycopene tended to pay a high premium price for functional foods. (b) Food neophobia had a direct negative effect on consumers’ attitudes toward adopting functional foods.	(a) Psychological characteristics (knowledge). (b) Psychological characteristics (food neophobia)	Willingness to pay
Labrecque et al. [89]	2006	Survey	Eggs with Omega-3, milk with calcium, and orange juice with calcium	North America	545	(a) French Canadian students had positive attitudes toward functional foods compared to French students who trusted the health information on functional foods less. (b) Believing in the credibility of information positively affected consumers’ functional food acceptance. (c) A high level of knowledge positively influenced consumers’ functional food acceptance. (d) Food neophobia was negatively related to consumers’ attitudes toward functional foods.	(a) Socio-demographic characteristics (geography and nationality). (b) Psychological characteristics (beliefs). (c) Psychological characteristics (knowledge). (d) Psychological characteristics (food neophobia)	Purchase intention
Landström et al. [86]	2007	Survey	Seven functional food items	Europe	972	(a) Well-educated consumers had a greater intention to purchase functional foods. (b) Consumers who tended to adopt healthy behavior (i.e., taking nutraceuticals, taking dietary supplements) were more likely to accept functional foods. (c) Consumers’ beliefs in the health effect of functional foods were positively correlated to their functional food acceptance. (d) Swedish consumers who had a diet-related problem were likely to consume cholesterol-lowering functional foods.	(a) Socio-demographic characteristics (education). (b) Behavioral characteristics (health related behavior). (c) Psychological characteristics (beliefs). (d) Physical characteristics (presence of diet-related problem)	Functional food consumption
Lu [56]	2015	Experimental study	The descriptions of 30 hypothetical functional foods (six carriers*five functional ingredients)	North America	Study 1 = 62; Study 2 = 93	(a) Consumers who cared about carrier–ingredient fitness were more willing to purchase functional foods. (b) Knowledge was a moderator between consumers’ perception of the carrier–ingredient combination and their purchase intentions.	(a) Product characteristics (carrier/ingredient combination). (b) Psychological characteristics (knowledge)	Purchase intention
Lyly et al. [66]	2007	Experimental study	B-glucan soup	Europe	1157	(a) Consumers were unwilling to compromise on the taste for health benefits.	(a) Product characteristics (taste)	Use of functional foods
Marette et al. [70]	2010	Experimental study	Yoghurts with added plant sterols	Europe	97	(a) Information that details the health benefits of cholesterol had a positive influence on consumers’ willingness to pay.	(a) Product characteristics (health information)	Willingness to pay
Markosyan et al. [73]	2009	Survey	Apples with a coating that contains specific flavonoids and antioxidants	North America	730	(a) Information about the potential health benefits of antioxidants positively influenced consumers’ willingness to pay for functional foods. (b) Consumers living in Seattle were less likely to pay a premium for functional products compared to consumers living in Spokane.	(a) Product characteristics (health information). (b) Socio-demographic characteristics (geography)	Willingness to pay
Markovina et al. [40]	2011	Survey	Functional food concept	Europe	1035	(a) Consumers between the ages of 19 and 30 were inclined consume functional food; female consumers living in a smaller household with high incomes were willing to purchase functional foods. (b) Health awareness, trust, and perceived price influenced young Croatian consumers’ attitudes toward functional food.	(a) Socio-demographic characteristics (age, gender, size of household, and income). (b) Psychological characteristics (attitude)	Willingness to buy
Melbye et al. [112]	2015	Experimental study	Milk-based meal replacement drink	Europe	100	(a) The feature of functional energy drink advertising influences the product credibility for consumers and product consumption. If consumers communicate through a person with lean figure (sportier, leaner), consumers consider the health benefits more credible.	(a) Psychological characteristics (trust)	Purchase intention
Mirosa & Mangan-Walker [61]	2018	Mixed methods	Juice; milk with added calcium; muesli bar with added protein and vitamin D	Oceania	193	(a) Chinese consumers were not willingness to pay more than 40% extra for functional foods. (b) Brand highly influenced consumers’ choice of functional foods. (c) Consumers who placed great importance on their mobility health were more willing to purchase functional foods to prevent mobility-related illnesses. (d) Consumers trusted information about functional foods advertised or publicized through authoritative figures.	(a) Product characteristics (price). (b) Product characteristics (brand). (c) Psychological characteristics (motivations). (d) Psychological characteristics (trust)	Willingness to purchase
Moons et al. [65]	2018	Survey	Spirulina-enhanced food	Europe	1325	(a) Health consciousness and taste were major determinants of consumers’ functional foods adoption. (b) Food neophobia negatively influenced foodies’ functional food adoption but not that of sporting individuals or vegetarians.	(a) Psychological characteristics (health consciousness). (b) Products characteristics (taste). (c) Psychological characteristics (food neophobia)	Functional food adoption intention
Moro et al. [77]	2015	Choice experiment	A hypothetical yogurt with two functional attributes (probiotics and catechin enrichment)	Europe	600	(a) Consumers between the ages of 45 and 64 were willing to pay for catechin-enriched yogurt; female consumers had slightly higher intentions to pay for catechin-enriched yogurts; consumers’ who completed the middle and tertiary educational levels, who were married or widowed, who were part of the second lowest and second highest income brackets, and who lived in a larger household reported a higher willingness to pay for functional foods. (b) Consumers’ health status (BMI) may be related to their willingness to pay for catechin-enriched functional foods. (c) Consumers’ lifestyle could influence their willingness to pay for functional foods.	(a) Socio-demographic characteristics (i.e., age, gender, educational level, marital status, income, and household size). (b) Physical characteristics. (c) Behavioral characteristics (lifestyle)	Willingness to pay
Narayana et al. [50]	2020	Survey	Functional dairy product	Asia	307	(a) Consumers from Sri Lanka were concerned about the price of products rather than their health benefits. (b) Taste was one of the most important motives for functional food consumption among Sri Lankan consumers.	(a) Product characteristics (price; taste)	Functional food choice
Nguyen et al. [100]	2020	Survey	Functional yogurts	Asia	596	(a) Subjective norm was positively correlated with consumers’ intention to purchase functional yogurts. (b) Health consciousness influenced consumers’ attitudes and was a significant determinant of consumers’ willingness to use functional foods. (c) The perceived price of functional yogurts had a negative influence on consumers’ purchase intentions.	(a) Psychological characteristics (motivation). (b) Psychological characteristics (health consciousness). (c) Product characteristics (price)	Purchase intention
Nystrand & Olsen [97]	2020	Survey	Milk and other dairy products with added vitamin D	Europe	810	(a) Norwegian consumers’ attitudes toward eating functional foods were positively influenced by utilitarian values and negatively influenced by hedonic values. (b) Consumers’ self-efficacy and social pressure were important motivators for their consumption of functional foods.	(a) Psychological characteristics (attitude). (b) Psychological characteristics (motivations)	Purchased intention
Ozen et al. [43]	2013	Survey	Skimmed milk, fiber-rich bread/cookies, probiotics, breakfast cereals and tea with functional components	Europe	1386	(a) Female consumers preferred consuming soymilk, fiber-rich bread/cookies, and tea, whereas male consumers preferred consuming functional breakfast cereals; consumers’ consumption of functional foods was significantly correlated with their increasing age; consumers who completed a medium education level preferred consuming fiber-rich bread/cookies; consumers who had a medium income preferred to consume breakfast cereals. (b) Physically active consumers were likely to consume soymilk, breakfast cereals, probiotics, and red wine, whereas obese consumers were less inclined to use breakfast cereals and fiber-rich bread/cookies.	(a) Socio-demographic characteristics (gender, age, educational level, and income). (b) Behavioral characteristics (health-related behavior)	Functional food consumption
Pappalardo & Lusk [31]	2016	Experimental auction	A new functional snack made with white lupine and citrus fiber	Europe	156	(a) Consumers who believed in the values of functional foods and the benefits on their personal health were more likely to accept functional foods. (b) Consumers who were concerned about their health were willing to compromise on the price of functional foods for the health benefits.	(a) Psychological characteristics (Beliefs). (b) Psychological characteristics (health consciousness)	Willingness to pay
Patch et al. [105]	2005	Survey	Novel foods enriched with Omega-3 fatty acids	Oceania	129	(a) Consumers’ attitudes significantly influenced their intention to consume them	(a) Psychological characteristics (attitude)	Intention to consume
Peng et al. [88]	2006	Survey	CLA-enriched dairy products	North America	803	(a) Families with teenagers were more likely to purchase functional foods. (b) Consumers who perceived the health benefits of functional foods were likely to consume functional foods. (c) Consumers who had previously purchased functional foods were interested in purchasing functional foods.	(a) Socio-demographic characteristics (household standard). (b) Psychological characteristics (perceptions). (c) Behavioral characteristics (health-related behavior)	Consumer acceptance
Phuong & Dat [102]	2017	Survey	Functional yogurts	Asia	242	(a) Consumers with positive attitudes toward functional foods had higher purchase intentions. (b) Consumers with a higher level of social prestige were more likely to purchase functional foods.	(a) Psychological characteristics (attitude). (b) Psychological characteristics (motivation)	Purchase intention
Rezai et al. [82]	2012	Survey	Synthetic functional foods	Asia	439	(a) Young consumers were more interested in purchasing functional foods; consumers with a higher income level had higher purchase intentions toward functional foods. (b) Consumers who subscribed to cooking or health magazines, who were vegetarians, and who had experience working for a food production company were more aware of functional foods.	(a) Socio-demographic characteristics (age, income). (b) Behavioral characteristics (lifestyle)	Purchase intention
Rezai et al. [101]	2014	Survey	Synthetic functional foods	Asia	2004	(a) Subjective norms had a positive effect on consumers’ intentions to accept synthetic functional foods. (b) Consumers who perceived the benefits of functional foods tended to accept synthetic functional foods.	(a) Psychological characteristics (motivation). (b) Psychological characteristics (perceptions)	Intention to purchase
Saba et al. [35]	2010	Conjoint study	Cereal-based products or non-cereal products containing beneficial compounds from grains	Europe	2392	(a) The effect of consumers’ perceptions about the health benefits of health information on their likelihood to buy functional foods differed across different European countries (i.e., Finland, Germany, Italy, and the UK).	(a) Product characteristics (health information)	Likelihood to buy
Sandmann et al. [91]	2015	Mixed methods	Vitamin D-fortified food	Europe	1051	(a) Health awareness had a positive effect on consumers’ acceptance of vitamin D-fortified food. (b) Consumers trusted professional health care organization as credible source of information.	(a) Psychological characteristics (health consciousness). (b) Psychological characteristics (trust)	Consumer acceptance
Schnettler et al. [78]	2015	Survey	Functional food concept	South America	400	(a) Consumers’ educational level, socio-economic status, and the presence of children influenced their functional food acceptance. (b) Consumers’ knowledge affected their functional foods acceptance.	(a) Socio-demographic characteristics (educational level, socio-economic status, and presence of children). (b) Psychological characteristics (knowledge)	Willingness to purchase
Shan et al. [111]	2017	Survey	Enriched processed meat	Europe	486	(a) Consumers were uncertain and negative about the health benefits of enriched processed meat products.	(a) Psychological characteristics (trust)	Purchase intention
Siegrist et al. [75]	2008	Survey	Functional food concept	Europe	248	(a) Older consumers were the primary consumers of functional foods. (b) Consumers were more inclined to purchase functional foods with physiological health claims compared to psychological health claims. (c) Consumers who trusted the food industry tended to accept functional foods.	(a) Socio-demographic characteristics (age). (b) Product characteristics (health information). (c) Psychological characteristics (trust)	Willingness to buy
Siegrist et al. [27]	2015	Survey	Four functional foods carriers with functional health benefits statements	Europe	945	(a) Chinese consumers showed a higher purchase intention toward functional foods than Germans. (b) Consumers who were more trusting of the food industry were willing to buy functional foods. (c) Food neophobia had a negative effect on consumers’ willingness to buy functional foods among Chinese consumers, whereas it did not influence German consumers. (d) Health benefits claims on functional food products increased Chinese consumers’ willingness to buy them.	(a) Socio-demographic characteristics (nationality). (b) Psychological characteristics (trust). (c) Psychological characteristics (food neophobia). (d) Product characteristics (health information)	Willingness to buy
Stojanovic et al. [62]	2013	Survey	Four product categories	Europe	479	(a) Consumers’ level of knowledge (information) affected their frequency of functional food consumption. (b) Consumers’ household standard (accompanied by children) affected their frequency of functional food consumption; consumers who had a higher educational level and higher income tended to buy functional foods. (c) The perception of functional foods’ goodness (good/bad) influenced their frequency of functional food consumption. (d) A higher perceived price decreased consumers’ (good/bad) influenced their frequency.	(a) Psychological characteristics (knowledge). (b) Socio-demographic characteristics (household standard, education, and income). (c) Psychological characteristics (perceptions). (d) Product characteristics (price)	Functional food consumption
Szakály et al. [121]	2012	Survey	Functional food enriched with vitamins, minerals, low sugar, low fact, and higher fiber	Europe	1000	(a) Lifestyle and health behavior influenced consumers’ preferences for functional food products.	(a) Behavioral characteristics (lifestyle)	Functional food preferences
Szakály et al. [18]	2019	Survey	Probiotic (functional) yoghurt	Europe	500	(a) Consumers with higher educational levels and higher incomes were more willing to purchase functional foods. (b) Consumers who had more positive attitudes toward functional foods (i.e., believing functional foods’ health benefits) were more willing to pay a premium for functional foods.	(a) Socio-demographic characteristics (education, income). (b) Psychological characteristics (attitude)	Willingness to pay
Temesi et al. [28]	2019	Survey	28 functional food carrier/ingredient combinations	Europe	1016	(a) Consumers were unwilling to compromise on the taste of functional foods for health benefits. (b) The perceived correspondence of health effects and carriers-ingredients combinations positively influenced consumers’ functional food acceptance.	(a) Product characteristics (taste). (b) Product characteristics (carrier/ingredient combination)	Intention to buy
Urala & Lähteenmäki [7]	2004	Survey	Eight different types of functional foods concepts	Europe	1158	(a) Consumers’ attitudes toward the perceived reward from using functional foods and their confidence in functional foods were major determinants of their willingness to use functional foods.	(a) Psychological characteristics (attitude)	Willingness to use
Van Kleef et al. [57]	2005	Reanalyzed existing data	10 different health claims systematically combined with 10 different food carriers	Europe	50	(a) Consumers preferred margarine and yoghurt products as attractive carriers compared to chewing gum, ice cream, and chocolate. (b) Consumers preferred functional foods that communicate the health benefits of reducing the risk of physiologically based illnesses more than psychologically based illnesses.	(a) Product characteristics (carrier/ingredient combination). (b) Product characteristics (health information)	Intention to try
Vecchio et al. [98]	2016	Experimental auction	Omega-3-enriched mozzarella cheese	Europe	150	(a) Consumers were more willing to pay for Omega-3-enriched mozzarella if they believed in the health benefits of preventing cardiovascular and rheumatic diseases. (b) Consumers’ self-efficacy was an important motivator for their functional food consumption.	(a) Psychological characteristics (beliefs). (b) Psychological characteristics (motivation)	Expectations of functional food consumption
Verbeke et al. [58]	2009	Experimental study	Calcium-enriched fruit juice; Omega-3-enriched spread; fiber-enriched cereals	Europe	341	(a) Consumers preferred functional foods to have a healthier image and a natural combination of ingredients. (b) Consumers may prefer functional foods with health and nutrition claim compared to a reduction of disease risk claim. (c) Consumers’ purchase intentions were negatively influenced by the presence of children under the age of 12 and positively influenced by the presence of teenagers.	(a) Product characteristics (carrier/ingredient combination). (b) Product characteristics (health information). (c) Socio-demographic characteristics (household standard)	Purchase intention
Verbeke [79]	2005	Survey	Functional food concept	Europe	215	(a) The presence of an ill family member may increase consumers’ functional food consumption. (b) Consumers who believed the health benefits of functional foods were more likely to accept functional foods. (c) Consumers with a higher level of knowledge were less likely to accept functional foods.	(a) Psychological characteristics (health consciousness). (b) Psychological characteristics (beliefs). (c) Psychological characteristics (knowledge)	Consumer acceptance
Verneau et al. [32]	2019	Experimental auction	Canned tomatoes enriched with lycopene	Europe	100	(a) Older consumers and female consumers were more likely to consume functional foods. (b) Consumers with less knowledge about functional foods were more likely to buy functional foods after they received functional foods’ health benefits information. (c) Food neophobia had a direct negative effect on consumers’ attitudes toward adopting functional foods. (d) Consumers who trusted science were more willing to pay for functional foods. (e) There was a positive correlation between information about the benefit of lycopene and consumers’ willingness to pay for lycopene-enriched functional foods.	(a) Socio-demographic characteristics (age, gender). (b) Psychological characteristics (knowledge). (c) Psychological characteristics (food neophobia). (d) Psychological characteristics (trust). (e) Product characteristics (health information)	Willingness to pay
Wortmann et al. [29]	2018	Survey	Selenium-biofortified apples	Europe	356	(a) Consumers with a high school or university degree were less accepting of functional foods. (b) Perceived health effects increased consumers’ acceptance of functional foods.	(a) Socio-demographic characteristics (educational level). (b) Psychological characteristics (perceptions)	Consumer acceptance
Xin & Seo [103]	2019	Survey	Imported Korean functional foods	Asia	361	(a) Consumers’ positive attitude toward functional foods positively influenced their purchase intention. (b) Consumers’ perceived behavioral control positively influenced their purchase intentions. (c) Consumers’ subjective knowledge and health consciousness positively influenced their intention to purchase functional foods.	(a) Psychological characteristics (attitude). (b) Psychological characteristics (perceived behavioral control). (c) Psychological characteristics (knowledge). (d) Psychological characteristics (health consciousness)	Purchase intention
